# Preparation and properties of the specific anti-influenza virus transfer factor

**DOI:** 10.1186/1746-160X-6-22

**Published:** 2010-09-13

**Authors:** Chongbi Li, Lihua Huang, Yanping Wang, Xiangle Li, Shaowei Liang, Yingna Zheng

**Affiliations:** 1 Center of Biopharmceutical Engineering in Zhaoqing University, 526061, Zhaoqing City,Guangdong Province, PR China

## Abstract

Specific anti-influenza virus and normal transfer factors prepared in an experimental animal model, the pig, have been tested for their components, characteristics, and activity of known specificity. Two transfer factors are small molecular mixture which consist entirely or partly of polypeptides and polynucleosides. Moreover, the biological activity of transfer factors could be approved by Rosettes test and specific skin test. The study would lay a foundation for the research and development of other specific transfer factor.

## Introduction

Transfer Factor (TF) was discovered in the 1940's and has been extensively studied for the past 50 years (1). In recent years, it has been known that Transfer Factor can transfer cell-mediated immunity (CMI) from an immune donor animal to a non-immune recipient. And now it is not only a scientifically recognized delivery system for transferring immune system advantages from one species to another but also most effective in regulating immunity to infections in which cell-mediated immunity (CMI; T cells) is important for controlling the infection [1]. It has been studied in various types of infections including viruses, bacteria, and fungal organisms. Therefore, Many kinds of TFs derived from different animals are prepared and applied in clinic. The clinic practice showed that transfer factor is a material that also has the ability to modulate the immune system [2]. Moreover, Transfer Factor has been found to be extremely safe. Therefore, the products manufactured incorporating the process are anticipated by the industry expert to be the "next wave" of nutritional supplementation, operating in the newly defined area of "structure/function"[3].

Transfer factor, an immunomodulator of low molecular weight capable of transferring antigen-specific cell mediated immune information to T-lymphocytes, has been used successfully over the past quarter of a century for treating viral, parasitic, and fungal infections, as well as immunodeficiencies, neoplasias, allergies and autoimmune diseases. Moreover, several observations suggest that it can be utilized for prevention, transferring immunity prior to infection. Because it is derived from lymphocytes of immune donors, it has the potential to answer the challenge of unknown or ill-defined pathogens[4]. Thus, it is important that a specific TF to a new antigen can be made swiftly and used for prevention as well as for the treatment of infected patients. Such as influenza viruses infection presents a threat of producing a pandemic This is of great concern, since no effective vaccine is available or can be made before the occurrence of the event.

We present arguments for the use of cell mediated immunity for the prevention of the infection as well as for the treatment of infected patients [5]. Similarly, transfer factors that are obtained from a host that has been infected with a certain pathogen are pathogen specific. Although such preparations are often referred to in the art as being "antigen specific"due to their ability to elicit a secondary immune response when a particular antigen is present, transfer factors having different specificities may also be present. Thus, even the so called "antigen specific", pathogen specific transfer factor preparations may be specific for a variety of antigens.

Most of the original clinical trials with transfer factors [4,5] used parenteral injections to administer T.F. Obviously the oral route would be preferable, however, it was originally assumed that the acidic and enzymatic environment of the gastrointestinal tract would destroy the factors. Experimental and human trials have amply demonstrated there is little if any loss of transfer factor activity taken orally [6].

In this paper, we present the results of the methods of preparing and analyzing the specific transfer factor oral preparation in vitro experimental system. The data indicated that the methods were credible and the biological activity of the transfer factor resides entirely or partly in vitro.

## Materials and methods

### Raw material, apparatus, reagents

Pig spleens were obtained from slaughterhouse at Huanggang town in Zhaoqing city. Normal oral Transfer factors (H20013408) were bought from the drugstore in Zhaoqing city. Centrifuge (Avanti™J-30I, BECKMAN COULTER made in Japan), ultraspectrophotometer(UV-2550 made in Japan), central empty ultrafiltrition equipment (MOTIANMO company in Tientsin), tissue disintegrator, superlow temperature refrigerator(HETO UF 3410, Danmark) and other essential apparatus exist in our Lab. Some chemical reagents were bought from the chemical reagent store in Zhaoqing city.

### Pig vaccination

10 Healthy pigs weighted 75 kilogram were chosen and vaccinated with influenza vaccine for human use, every pig was injected through muscle one unit, and 15 days after the first injection, the second injection would be performed in the same dosage as the first time. In 20 days after the second injection, the pigs would be slaughtered, and the spleens would be collected and stored in refrigerator.

### Transfer factor preparation

Specific and non-specific transfer factor were prepared from the vaccinated and normal pigs spleens through superultrafiltrator equipped with a membrane of 6000 dalton. The method was referred to literature [6] and made some modifications.

These frozen pig spleens was taken turns through mechanically crashed, frozen and thaw reduplicatively, centrifugation, filtration, superultrafitration, formulation and finally an oral normal and specific transfer factors would be prepared for the characteristics examined. The unit would be confirmed according to the criteria from the seventh international session on TF, that is, OD (ABS)240-260 nm was up to 10 as one unit. And the TF oral solution was formulated with some excipients.

### Physicochemical and biological properties examination

#### Ultraviolet spectrum absorption

Two kinds of samples would be detected by ultraviolet spectrum absorption. Normal saline would be as a control. It would be considered qualified if the ratio of A_260/_A_280 _was larger than 1.8 after detecting.

#### Protein reaction

Protein would be detected with 20 percent of Sulfonic-Salicylic acid. It was considered as positive if the solution examined was cloudy whereas negative if lucidity.

#### Analysis of amino acids

Three milliter of the sample of Specific and non-specific TF were added eight percent of Sulfonic-Salicylic acid for three milliter respectively and put at quiescence for 40 min at 4°. And then permitted them centrifuging at 18000 g for 40 min. The samples were diluted and analysed with type of 835-50 Amino acid auto-analysing instrument.

#### The contents of polypeptide and nucleoside

Detection for content of polypeptides in TF including specific, non-specific and normal transfer factor sold on the medicine store would be performed by biuret reaction [3]. And the content of nucleosides in TFs would be examined by the method of phenylphenol reagent reaction according to standard curve drawn with the sample of RNA bought from the Sigma [3] and calculated through a formula as follows:

Content of nucleosides (ug/ml) equal to value from the standard curve multiply multiples diluted

#### Heat lability of the transfer factor

To test for the heat sensitivity of the transfer factor solution, aliquots were diluted 20-fold with 50 mM Tris, pH 8, and then incubated for 10 min at various temperatures, ranging from -20°to 80°C. The lability of TFs would be detected according to ultraviolet spectrum absorption. The different TFs were put under the different temperature (-20°, 4°, 37°, 60° and 80°) for 10 days.

#### Activity of specific transfer factor

Because transfer factor can make a mammalian immune system to elicit a secondary immune response, whereas infecting pathogen or antigenic agent to facilitate a secondary, or delayed type hypersensitivity, Thus the animals administered specific TF would be detected their delayed type hypersensitivity through skin test. 15 rabbits without any antigen infecting were chosen to divide 3 groups, 5 each group. Each rabbit in the first group would administered orally specific TF one unit once daily for 10 days, the second group would be orally given normal TF about dosage as above mentioned, the third group received the same amount of sterile saline diluent as control. And three days later the animal was shaved on back and injected intradermally with concentrations of 10 ul influenza vaccine and bcg vaccine respectively. Positive reaction would be considered according to the size of swollen nodus if it was larger than 1 mm. Contrarily, it was negative.

#### Germ examination

Germ in the preparations were check up in terms of method [7] whether the products contain aerobe, anaerobic, saprophyte and fungi.

### Toxic test for mice

15 healthy BALB/c mice were chosen for toxic test. And 3 groups were divided randomly, 5 mice in one group. The mice in group TF were infused with concentrated TF oral solution 40 units once daily for 7 days. Moreover, control group administering normal saline at the same dosage. Activation and appearance of the mice tested would be observed after administering high dosage TF with control group as comparation (Health Department of PR China, 1990).

### Activity of TF in vitro

E-rosettes test is an effective and simple method which identify the activity of T-cells from animals [6]. The idea had been accepted that the sheep erythrocytes could cluster around the T-cells and form rosettes (E-rosettes) that could be viewed and counted under a microscope. The test was to identify and separate white blood cells (2 × 10^6^/ml)from human by Ficoll-Conray centrifugation by mixing them with red blood cells (erythrocytes, concentration of 1%) from rat. And the action of peripheral lymphocytes of human was investigated. The concretive operations was followed as table 1 and repeated for 3 times.

**Table 1:** E-Rosettes Assay

Adding reagents	Test tube A	Test tube B	Control tube
Peripheral lymphocyte of human	0.05 ml	0.05 ml	0.05 ml
20% of calf serum protein formulated with Hank's solution	0.25 ml	0.25 ml	0.25 ml
Normal TF 6 kD (diluting 100 multiple)	0.25 ml	――	――
Specific TF 6 kD (diluting 100 multiple)	――	0.25 ml	――
0.9% Normal saline(N.S)	――	――	0.25 ml

mixed at 37°C incubating for 1 h, and centrifuged at 2500 g for 10 min, Then discarded supernatant and resuspended pellet.			

1% of rat erythrocytes	1 dript	1 dript	1 dript

After mixed and centrifuged at 500 g for 5 min, and then discarded supernatant and resuspended pellet to add one dript of 2.5% aldehyde and mixed to incubate for 15 min at 4°C after then put out to wipe blade, dye with Gimsas and counting.

## Results

Smaller molecular weight molecules (e.g., ultrafiltrations having molecular weights of about 6,000 D or less), including any transfer factor from the pig spleens, remained in solution. The physicochemical properties of TFs presented whatever specific or non-specific TF preparations were all transparency and light yellow fluid with a pH 6.5-7.0 and negative protein reaction. They contain sixteen amino acid residues without examining Ser (Table 2). These very small transfer factor molecules contain the essence of the immunological message.

**Table 2:** The contents of amino acid residues in Specific and non-specific TF

Amino acid(gross)	specific TF(mg/ml)	non-specific TF(mg/ml)
Asp	320.58	329.23
Thr	149.63	152.58
Ser	199.08	212.63
Glu	523.03	612.86
Gly	241.12	249.06
Ala	216.78	258.26
Cys-Cys	28.54	31.25
Val	178.23	182.06
Ser	-	-
Met	7.23	7.16
Ile	95.12	94.98
Leu	226.13	236.28
Tyr	92.26	89.78
Phe	105.86	116.45
Lys	236.21	225.23
His	63.28	58.23
Arg	86.12	79.86
Pro	146.23	162.83
NH_2_	65.23	70.98.

### Ultraviolet spectrum analysis

**Figure 1:**
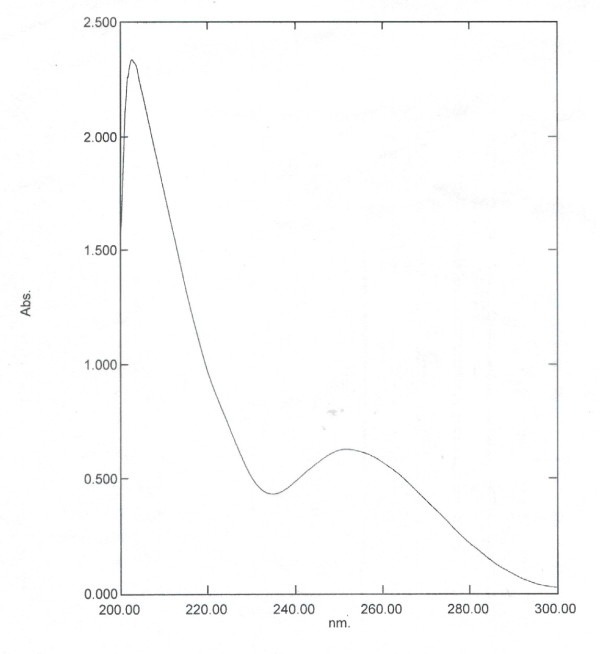
Non-specific TF product ultraviolet absorption light spectrogram.

**Figure 2:**
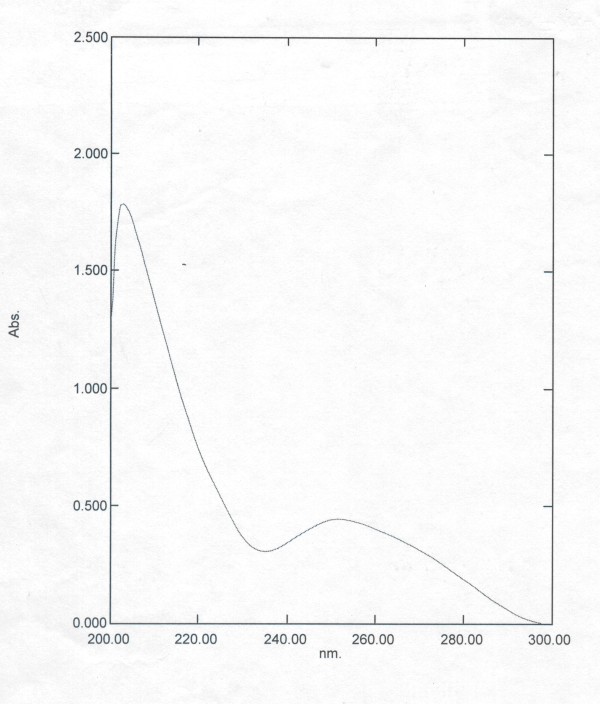
**Specific TF product ultraviolet absorption light spectrogram**.

The analysis of TF preparations in ultraviolet spectrum absorption indicated that the highest peak of specific and non-specific TF were similar without differences pertaining to the range of normal TF (Table 3 and Figure 1, 2). And the ratio of A260 to A280 was up to the criteria of National Health Department on transfer factor.

**Table 3:** Ultraviolet absorption spectrometry analysis comparison

*Subject*	*batch*	***A***_***260***_	***A***_***280***_	***A***_***260***_/***A***_***280***_	**A**_**max**_
Nonspecific TF (30 times)	1	0.354	0.156	2.269	11.43
	2	0.565	0.216	2.616	18.84
Specific TF (60 times)	1	0.405	0.203	1.995	26.46
	2	0.404	0.187	2.162	26.58
TF sales (20 times)	1	0.480	0.213	2.252	14.26

### Contents of polypeptides and nucleotides in TFs

**Figure 3:**
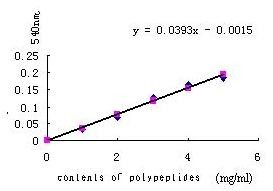
**Protein standard diagram of curves**.

**Figure 4:**
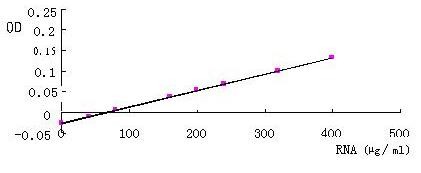
Nucleoside standard diagram of curves.

Detection for content of polypeptides in TF indicated that content of specific was higher than that of non-specific and was also closed to normal transfer factor sold on the store (Figure 3 and Table 4). And the content of ribonucleotides in TFs were also closed to normal TF sold in market (Figure 4 and Table 5).

**Table 4:** Poly-peptides content comparison

*subject*	*batch*	***ABS***_***540 nm***_	Polypeptide (mg/ml) (OD 10)
Non-specific TF	1	0.026	0.702
	2	0.025	0.681
Specific TF	1	0.043	1.130
	2	0.039	0.039
TF sold	1	0.038	1.000

**Table 5:** Nucleoside content comparison

*Subject*	*batch*	***ABS***_***670 nm***_	RNA (mg/ml) (OD = 10)
Non-specific TF	1	0.155	0.455
	2	0.094	0.303
Specific TF	1	0.169	0.489
	2	0.173	0.500
TF sold	1	0.102	0.323

Transfer factor is heat-sensitive. A solution of specific and non-specific transfer factor heated from 37 to 80° retained full biological Activity identified according to the varieties of TFs untravilet spectrum absorptions. When the TF solutions were at -20°C, 4°C and 20°C or heated from 37°C to 60°C retained partial biological activity (Table 6). But when they were heated to 80°C they were inactivated. And there were no differences between them (Table 6 and 7).

**Table 6:** Variation of ultraviolet spectrometry absorption in specific TF under different temperature preserving (to dilute 30 times) for 10 d

Conditions	**A**_**260**_	**A**_**280**_	**A**_**260**_**/A**_**280**_
Original	0.490	0.221	2.219
-20°C	0.477	0.212	2.246
4°C	0.506	0.229	2.210
20°C	0.509	0.236	2.158
60°C	0.531	0.280	1.896
80°C	0.651	0.392	1.661

**Table 7:** Variation of ultraviolet spectrometry absorption in non-specific TF under different temperature preserving (to dilute 30 times) for 10 d

Conditions	**A**_**260**_	**A**_**280**_	**A**_**260**_**/A**_**280**_
Original	0.408	0.180	2.269
-20°C	0.471	0.213	2.214
4°C	0.479	0.225	2.129
20°C	0.463	0.200	2.318
37°C	0.466	0.203	2.290
60°C	0.501	0.278	1.802
80°C	0.628	0.369	1.702

It was qualified for their having no aerobe, anaerobic, saprophyte and fungi through bacteriological checkup in few batch of TF products.

Toxic test for mice indicated that TF preparations including specific and non-specific TF were all non-toxic for after have been administered TF in large quantity dosage non of mice appeared abnormal and discomfortable even dying.

### Activity of specific transfer factor

**Figure 5:**
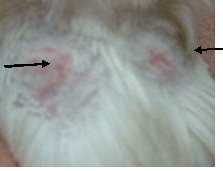
**Intradermal test from specific TF**. Arrowhead pointed skin test result with influ vaccine on the left, another arrowhead pointed that with bcg vaccine

**Figure 6:**
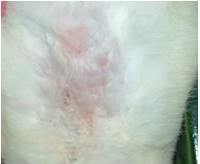
**Intradermal test from non-specific TF**. Arrowhead pointed skin test result with influ vaccine above the figure, another arrowhead downwards pointed that with

The skin test showed that the greater increase in size, or swelling, of the back skin reaction (increases of 3.0 mm to 5.0 mm) over that of the control rabbits (Figure 5 and 6, increases of 0.5 mm and 1 mm, respectively) and indicated that the influenza specific pig transfer factor containing solution induced a delayed type hypersensitivity reaction in the back skin within about twenty four hours following the introduction of the influenza virus vaccine.

### Activity of TF in vitro

**Figure 7:**
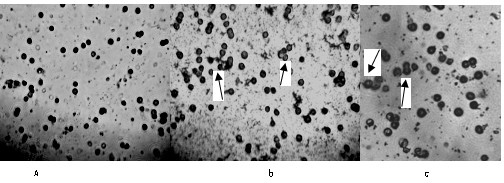
**E-Rosettes formation of samples**. A. Control EAC B. Non-specific EAC C. Specific EAC

E-rosettes test showed that TF could promote human lymphocyte to form E-rosettes with sheep erythrocytes. However, in our study, it is founded that TF could also promote human lymphocyte to form E-rosettes with rat erythrocytes. Moreover, the action of TF (10(-1) -10(-3) U) upon active rosette formation was studied to quantify T cells could significantly increase EAC percentage, but no significant difference between specific and non-specific TF(P < 0.01, Table 8 and Figures 7a-c).

**Table 8:** Comparison of E-Rosettes formation on TFs

Batch of TF	Control%	Normal TF 6000 × 10(-2)		Specific TF6000 × 10(-2)		Increasing EAC percentage
		E-rosettes%	difference%	E-rosettes%	difference%	
1	21	34	13	31	10	3
2	23	40	17	34	11	6
3	18	38	20	33	15	5
Average	20.7	37.4	16.7	32.7	12	4.7

## Discussion

In this experiment, specific anti-influenza virus TF was prepared by a untrafiltrative method with a septum of molecular weight 6000. However, early researchers prepared T.F. from leukocyte extracts of donors through dialysis. The specific T.F. was qualified for its preparing criteria on the characteristics including physico-chemical and biology activity. It had been prepared from the vaccinated donor pig spleen cells, and the preparing procedure was not only simple but also the quality of product was higher. Particularly, the oral preparation is convenient to users.

The simplest interpretation of the data is that transfer factor is a small polypeptide and ribonucleotide molecule. Typically, transfer factor includes an isolate of proteins obtained from immunologically active mammalian sources and having molecular weights of less than about 10,000 daltons [1,2]. In our study, the component of transfer factor functions in is that small molecular mixture including polypeptide with molecular weight lower than 6000 daltons. Whether these complex mixture represent different functions respectively remains unknown. But it can assume that these different components maybe an inducer component, antigen specific component, and a suppressor component. Since our immune system is one of our defenses against disease. It is the bodys actual agent involved in healing or recovering from an illness. And transfer factor could enable the T cells of our immune system to set off immediate alarms when certain antigens are identified as undesirable [7], and we know that there are the T inducer and T suppressor cells in our immune systems to an infecting pathogen or antigenic agent to facilitate a secondary, or delayed type hypersensitivity. Additionally, it was reported that the antigen specific region of the antigen specific transfer factors had been comprised about eight to about twelve amino acids and a second highly conserved region of about ten amino acids and thought to be a very high affinity T cell receptor binding region, and the nucleoside portion may be part of the inducer or suppressor fractions of transfer factor. Therefore, transfer factor, on a much smaller molecular weight scale, appears to be hypervariable and is adapted to recognize a characteristic protein on one or more pathogens [8].

Most of the original clinical trials with transfer factors [4,5] used parenteral injections to administer T.F. Obviously the oral route would be preferable, however, it was originally assumed that the acidic and enzymatic environment of the gastrointestinal tract would destroy the factors. Some human trials [4] have amply demonstrated there is little if any loss of transfer factor activity taken orally. Oral administration of transfer factor to mammals is supported by the fact that mammalian mothers supply transfer factor to their newborn children by way of colostrum, which the newborns ingest orally. Transfer factor survives the conditions of both the stomach and the small intestine, where transfer factor is absorbed into the bloodstream of the mammalian newborn. Thus, transfer factor is known to survive the intestinal tracts of mammals.

A fact of that the influenza specific pig transfer factor induced a delayed type hypersensitivity can interpret the multiple combinatorial patterns between these amino acids and nucleotides possibly create a vast number of different T.F. molecules. Such a large number of molecules would then satisfy the notion that a specific T.F. molecule is necessary to transfer immunity to each and every specific antigenic determinant [9]. Since three different TF components were no significant differences. Another words, T.F. transfers immune power to a recipient who will subsequently gain specific immunity.

It is known that transfer factor, when added either in vitro or in vivo to human immune cell systems, improves or normalizes the response of the recipient human immune system from the result of Rosette-test. It is known that the sheep cells attached themselves to certain cell-surface proteins that were characteristic of a subtype of T-cells called the T-helper cell. However, in our study, another assumption eventually emerged that the rat cells also could attach themselves to certain T cell-surface. Although the transfer factor phenomenon is described here in terms of one experimental system, the differentiating leukocyte, it might have further implications in developmental biology. Perhaps other types of cell-cell interactions leading to differentiation also involve the transmission of information by a small molecule such as transfer factor.

## Conclusions

Transfer factor has been obtained from a wide variety of other mammalian sources including mice, rabbits, pigs, cows, and other mammals. In addition, specific transfer factors have been generated against a single pathogen cell cultures or antigenspecific tissue-spleen, they have specificity for a variety of antigenic sites of that pathogen. Thus, these transfer factors are said to be " antigenspecific ".Similarly, transfer factors that are obtained from a host that has been infected with a certain pathogen are pathogenspecific.

Transfer factors are another noncellular part of a mammalian immune system with a molecular weight in about 6,000 Daltons (D) including polypeptides of may amino acids components and a nucleoside portion.

The specific pig transfer factor has the ability to generate an early secondary immune response in mammals as it could initiate an early delayed type hypersensitivity immune reaction in rabbit. However, it is clear that an appropriate in-vitro laboratory evaluation of each TF batch and of its destined recipient is essential in order to define the function and applications of the TF. Thus, what this suggests is that the transfer factor can not only use in treat influenza, but also prevent future breakouts as well for further study. This same action may apply to other viral infections like chronic fatigue and bronchitis.

## Competing interests

The authors declare that they have no competing interests.

## Authors' contributions

LH participated in the examination of STF, YW joined the preparation of STF, XL participated the examination of STF, SL and YZ also joined working on examinations of STF.

All authors have read and approved the final manuscript.

## References

[B1] Kirkpatrick CH Structural nature and functions of transfer factors Ann N Y Acad Sci 1993 685 362 8 23 10.1111/j.1749-6632.1993.tb35889.x 8363241

[B2] Kirkpatrick CH Activities and characteristics of transfer factors Biotherapy 1996 1 13 16 Alvarez-Thull L, Kirkpatrick CH. Profiles of cytokine production in receipients of transfer factor. Biotherapy 1996, 9, 55-59 10.1007/BF02628651 8993752

[B3] Chongbi Li Wang Wen Yuexian Qing Zou Zhaofen Comparison of the biological effects of PSBr-TF, PS-TF and Thymic Hormone Current Research in Transfer Factor 1993 China Science & Technology Press. Beijing 195 2012

[B4] Chang JJ Present research situation and clinical practice progress on transfer factor J Qinghai Univ (Nat Sci) 2007 25 2 31 35

[B5] Lawrence HS Borkowsky W Transfer Factor--current status and future prospects Biotherapy 1996 9 1 3 Chen Junhui, Tao Li, Li Jun et al., Laboratory Manual for Biochemistry, Science Publishers, Beijing, 2003 10.1007/BF02628649 8993750

[B6] Chongbi Li Tianqi Fang Jingqiu Zhang Immunologic Activity of Transfer Factor in oral and injecting administration in vitro and in vivo J Chinese Bioproducts 1997 10 91 934

[B7] The Committee of Bioproducts Criteria of the People's Republic of China Requirements for Biological Products 2000 Beijing: Chemical Industry Press 45 482

[B8] Kirkpatrick CH Transfer factors: identification of conserved sequences in transfer factor molecules Molecular Medicine 2000 6 332 341 10949913 PMC1949950

[B9] Alvarez-Thull L Kirkpatrick CH profiles of cycokine production in recipients of transfer factors Biotherapy 1996 9 13 55 59 10.1007/BF02628657 8993758

